# New insights into the gut microbiome in loggerhead sea turtles *Caretta caretta* stranded on the Mediterranean coast

**DOI:** 10.1371/journal.pone.0220329

**Published:** 2019-08-14

**Authors:** Vincenzo Arizza, Luca Vecchioni, Santo Caracappa, Giulia Sciurba, Flavia Berlinghieri, Antonino Gentile, Maria Flaminia Persichetti, Marco Arculeo, Rosa Alduina

**Affiliations:** 1 Department of Biological, Chemical and Pharmaceutical Sciences and Technologies (STEBICEF), Università degli Studi di Palermo, Palermo, Italy; 2 Istituto Zooprofilattico Sperimentale della Sicilia, Palermo, Italy; Universita degli Studi della Tuscia, ITALY

## Abstract

*Caretta caretta* is the most common sea turtle species in the Mediterranean Sea. The species is threatened by anthropomorphic activity that causes thousands of deaths and hundreds of strandings along the Mediterranean coast. Stranded turtles are often cared for in rehabilitation centres until they recover or die. The objective of this study was to characterize the gut microbiome of nine sea turtles stranded along the Sicilian coast of the Mediterranean Sea using high-throughput sequencing analysis targeting V3–V4 regions of the bacterial 16S rRNA gene. Stool samples were collected from eight specimens hosted in the recovery centre after a few days of hospitalization (under 7) and from one hosted for many weeks (78 days). To better explore the role of bacterial communities in loggerhead sea turtles, we compared our data with published fecal microbiomes from specimens stranded along the Tuscan and Ligurian coast. Our results highlight that, despite the different origin, size and health conditions of the animals, Firmicutes, Bacteroidetes and Proteobacteria constitute the main components of the microbiota. This study widens our knowledge on the gut microbiome of sea turtles and could be helpful for the set up of rehabilitation therapies of stranded animals after recovery in specialized centres.

## Introduction

The gut microbiota represents the ecological community of the microorganisms that reside in the gastrointestinal tract and influence host physiology, immunity and development in all animals studied so far [1]. In recent years studies of the complex microbial communities have rapidly been increased and have been facilitated by high throughput approaches based on next-generation sequencing of 16S rDNA [2]. Numerous studies demonstrated that the microbial genome (microbiome) is about 10–100 times larger than the host genome and that microbial enzymes are involved in numerous biological processes, such as energy production and food digestion [3–8].

In the last decade, the study on gut microbiota has also been extended to wild animals in order to determine the relationships between the microbiota and the diet, the environment and the host ecology and to understand pathogen transmission [1]. The gut microbiota was studied in many vertebrates, including birds [9,10], fish [11], amphibians [12], and reptiles [13–17]. It has been discovered that the microbiota plays a role in digestion homeostasis, general metabolic regulation and defence against pathogenic organisms in fish and birds [18,19].

The carnivorous loggerhead sea turtle (*Caretta caretta L*.) is currently considered “Vulnerable” by IUCN (https://www.iucnredlist.org/species/3897/119333622). Many events, such as incidental catches by fishing [20], water pollution [21], and global climatic changes, affect the health status of sea turtles [22] causing eventual stranding of these animals. Stranded sea turtles are usually recovered and hosted in recovery centres, and released back to the sea after rehabilitation [20].

To date, little is known about the gut microbiome diversity in the loggerhead sea turtle. The knowledge is limited to two recent studies [23,24]. The first one analysed microbiome from four fecal samples from three specimens and six cloacal samples from other five individuals stranded or accidentally caught along the coast of Tuscany and Liguria regions (Tyrrhenian Sea) [25]. The second study reported the fecal microbiome of twenty-nine sea turtles stranded or captured in trawling nets in the upper-west part of Adriatic Sea [26]. These two studies found a different microbial composition; in the first case the phyla more represented were Firmicutes, Proteobacteria and Bacteroidetes [23] while in the second one Firmicutes and Fusobacteria [24]. So far, more in-depth studies have been carried out on the herbivorous green turtle *Chelonia mydas* [25–28]. Besides the gut microbial composition, studies on the green turtles revealed that gut microbiomes differ between wild and stranded turtles [26] and after rehabilitation in recovery centres [27]. In addition, gut microbiome responds to shifts in habitat and diet in developing sea turtles [25] and it is acquired soon after settlement in the coastal waters [28].

The objective of this study was to investigate and to enlarge knowledge on the role and importance of the gut microbiome diversity in the loggerhead sea turtles stranded along the Sicilian coasts. In addition, our results were combined with data from the sea turtles stranded along the Tyrrhenian Sea coast in Tuscany and Liguria regions (Italy) [25] for a more complete data coverage.

## Material and methods

### Sample collection

Stool samples were collected from nine specimens of loggerhead sea turtle stranded along the coast of Sicily, Mediterranean Sea. The locations of the studied individuals were mapped using the QGIS software v. 2.18.2 (http://www.qgis.org) and are reported in Table 1. Specimens were conferred to the personnel of the Regional Centre of the Recovery for Sea Turtles at the Veterinary Public Health Institute of Sicily (IZS Sicily), located in Palermo; they are engaged in the recovery and transportation of loggerhead turtles to the Centre. The health status of the stranded turtles was assessed by an expert veterinary technician. Morphometric data such as sex, body weight and curved carapace length (CCL) were recorded and are presented in Table 1. During hospitalization, all sea turtles were housed separately in individual tanks with sea water. Tanks had been previously cleaned and disinfected with regular bleach. Every two days, tanks were cleaned and water was replaced. Duration of hospitalization in the Centre at the sampling date is listed in Table 1. In the Centre, turtles were fed twice a week with small pelagic fishes. Since the microbiome of animals from rescue centres might be highly biased, as demonstrated in the green sea turtle *C*. *mydas* [27] we proceeded with analysing the first fecal samples collected a few days after animal recovery. After collection, the fecal samples were stored at -20°C, until DNA extraction. The fecal sample (S5) is derived from a loggerhead sea turtle after rehabilitation just before being released back to the sea.

**Table 1:** Details of sea turtles and sampling. Geographical coordinates are expressed as decimal degrees (Map Datum: WGS84).

Sample	Sex	CCL^1^	Weight (Kg)	Stranding location	Latitude (N)	Longitude (E)	Recovery date	Sampling date	Hosp days^2^
S1	F	50	17	Terrasini (Palermo)	38.1603	13.0845	17/08/2017	24/08/2017	7
S2	F	61	21	Augusta (Siracusa)	37.2428	15.2287	20/08/2017	22/08/2017	2
S3	F	46	11	Pantelleria (Trapani)	36.8325	11.9344	08/09/2017	13/09/2017	5
S4	M	48	15	Porto Rosso (Catania)	37.5133	15.1060	07/08/2017	14/08/2017	7
S5	F	30	2.6	Catania	37.4852	15.0877	28/08/2017	14/11/2017	78
S6	F	51	7.4	Catania	37.4852	15.0877	30/07/2017	06/08/2017	7
S7	F	46	8	Catania	37.4852	15.0877	30/09/2017	02/10/2017	2
S8	F	71	29	Augusta (Siracusa)	37.2428	15.2287	26/08/2018	07/09/2018	7
S9	F	57	19	Pozzallo (Ragusa)	36.7202	14.8333	02/07/2018	04/07/2018	2

^1^ indicates the Curved Carapace Length.

^2^ indicates days of hospitalization before fecal sample collection.

### Ethics statement

All methods and experimental protocols on sea turtles were conducted by the personnel of the Regional Centre of the Recovery for Sea Turtles at IZS Sicily, in strict accordance with the recommendations of the Region of Sicily and the Ministry of Health (regional law n. 6067/2013 and national law n. 96/2016). All efforts were made to minimize animal suffering.

### Genomic DNA extraction, PCR amplification and sequencing

DNA was extracted from all the samples as described below. Each fecal sample was incubated in 3 ml of STE buffer (100 mM NaCl, 10 mM Tris-Cl, pH 8.0, 1 mM EDTA) containing 3-mm sterile glass beads for 1h at 70°C with periodic vortexing. After addition of 10 mg of lysozyme (Sigma-Aldrich), the samples were further incubated at 37°C for 1 h. 200 μl of 0.5 mg/ml Proteinase K and 600 μl of 10% SDS were added and the samples were incubated at 55°C for 90 minutes. 2 ml of 5 M NaCl were added and samples were mixed by inversion. After addition of 5 ml of chloroform, the samples were mixed by inversion for 30 minutes at RT. Samples were then centrifuged at 4500×g for 15 minutes at 4°C. The supernatant was transferred to a fresh tube and 0.6 volumes of isopropanol were added. Samples were then centrifuged at 13000 ×g for 30 minutes at 4°C. The supernatant was aspirated and discarded and the DNA pellet washed several times with 70% ethanol and resuspended in 1 ml di TE (10 mM Tris-Cl, pH 8.0, 1 mM EDTA). Purity and quantity of DNA were assessed via spectrophotometry (Nanodrop, Thermo Fisher Scientific, Waltham, MA). The extracted DNA was sent to Biodiversa srl, Rovereto (TN) for DNA sequencing of the V3-V4 region of the 16S rDNA using the primers described in Takahashi et al. 2014 [29] in one 300-bp paired end run on an Illumina MiSeq platform.

### Raw data processing and statistical analyses

Raw sequences were analysed following the UPARSE pipeline as previously described [30,31]. Using the USEARCH algorithm [32] several steps were made in order to remove low-quality reads that can generate errors in downstream analyses, merge the read-pairs and remove singletons before the OTU (Operation Taxonomic Units) clustering step, which was performed using an identity threshold of 97%. Moreover, chimeras were detected and removed by UPARSE during the clustering step (“cluster_otus” command). A total of 725157 filtered reads of all sample of *C*. *caretta* passed a quality filtering (71.24% of total reads). UPARSE pipeline was chosen for the higher resolution of the data in terms of contents of filtered reads and detected OTUs in respect to the QIIME pipeline [33] (Table 2 and S1 Table). Finally, from each OTU cluster, a single representative sequence was selected and used for taxonomical identification by SINA classifier on the latest SILVA dataset available when the analysis was performed [34] (https://www.arb-silva.de/ngs/). Rarefaction analysis was carried out plotting the number of observed OTUs against the total number of filtered reads for each sample. To evaluate the variations among samples, we analysed the dataset using Bray–Curtis distance matrix, which were visualized by principle coordinate analysis (PCoA). The analyses were performed with PRIMER 6+PERMANOVA software package from Plymouth Marine Laboratory, UK. Alpha diversity, Abundance-based Coverage Estimator (ACE), Chao1, Shannon-Wiener diversity, H’, and Simpson index, 1-D (this index takes values between 0 and 1), and evenness, e (equitability assumes a value between 0 and 1 with 1 being complete evenness), were estimated to determine the specific fecal microbial richness and diversity. Good’s coverage was estimated to evaluate the completeness of sampling. To enlarge the number of samples, sequences of *C*. *caretta* microbiota from sea turtle feces obtained by Abdelrhman *et al*. [23] were added in the analysis. T1 and T3 came from the same sea turtle after 40 and 37 days of hospitalization before sampling, T11 and T12 from different turtles after 28 and 41 days. Unfortunately, data comparison with Biagi *et al*. [26] was not possible due to the different data format and because different pipelines were applied: UPARSE in Abdelrhman *et al*. [23] and this study, and QIIME in Biagi *et al*. [24].

**Table 2:** Total number of OTUs resulting from the UPARSE pipeline dataset.

Sample	Total Reads	Merged Reads (%)	Filtered Reads	Chimeras	OTUs
S1	99926	72.65	68028 (93.7%)	263	91
S2	129304	78.26	93264 (92.2%)	358	149
S3	166807	76.71	120147 (93.9%)	570	153
S4	102189	71.35	67464 (92.5%)	92	89
S5	130991	76.79	94073 (93.5%)	301	116
S6	140362	79.2	102303 (92%)	573	188
S7	144340	75.60	101607 (93.1%)	470	234
S8	48370	72.71	36095 (75%)	736	206
S9	55625	74.98	42176 (76%)	1831	197
Total					1423

### Links to deposited data

The sequence dataset was deposited in the GenBank database (Bioproject PRJNA481425, Submission ID: SUB4304187). The sequence dataset can be downloaded and freely used for research purpose by users that are requested to acknowledge us and to cite this paper as reference to the data. Sequences will be available and downloaded after the acceptance of the paper.

## Results

### Sequencing output and analysis

**Fig 1:**
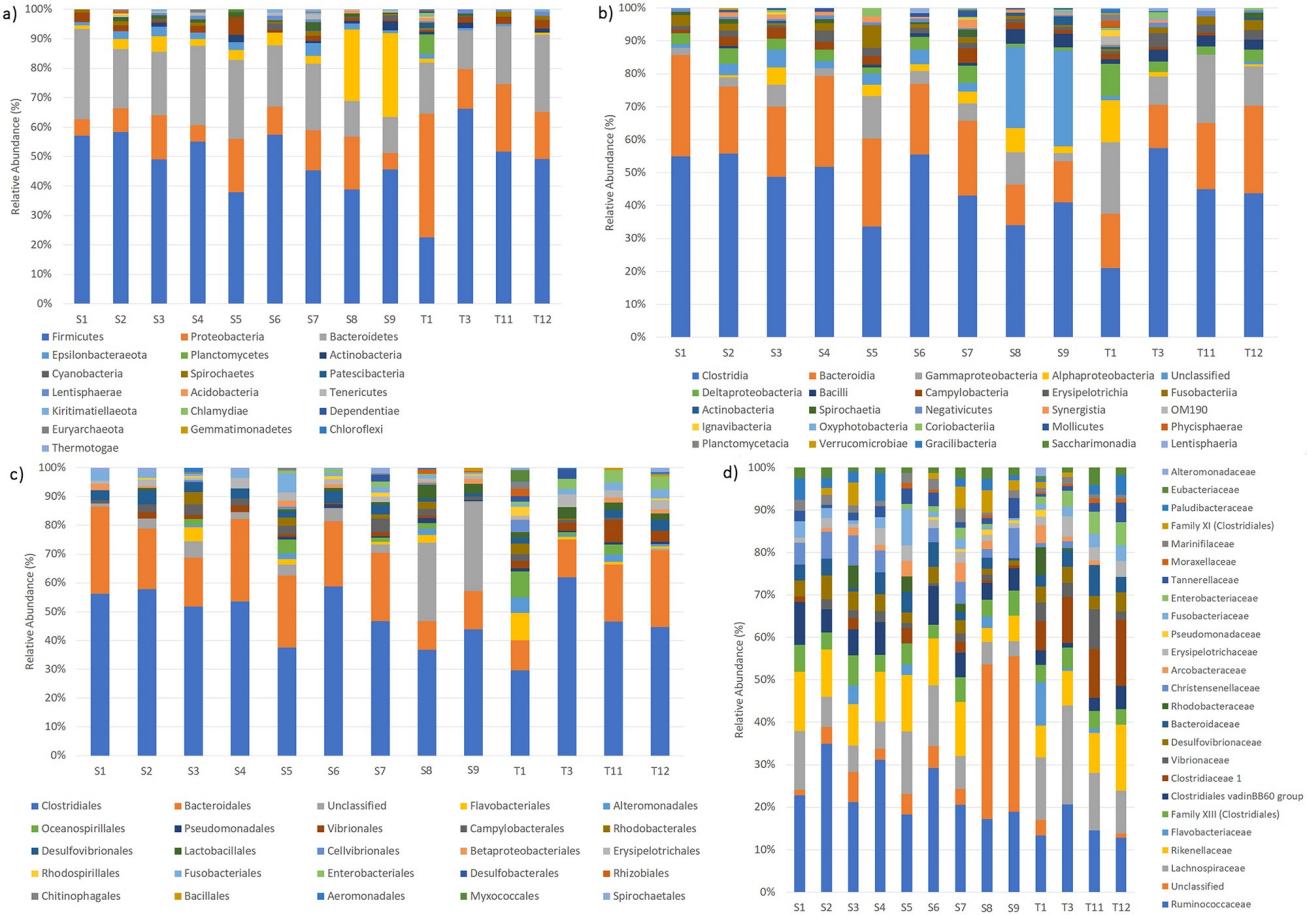
Relative abundance (%) of fecal bacterial communities in loggerhead sea turtles at different taxonomic levels. Microbial composition was determined taking into account only the 25 most abundant components of phylum (a), class (b), order (c) and family (d).

In total, 725157 high-quality reads (Q>33 and 470 bp in size) were filtered from 1017914 raw reads obtained from nine fecal samples (indicated by S). 1,423 unique OTUs were successfully identified using UPARSE pipeline (Table 2) and classified at family level using a 97% sequence similarity threshold against the “Silva” database (Fig 1). OTUs that were unable to be assigned were categorized as “Unclassified”. Each sample contained between 89 and 234 OTUs for a total of 1,423 that allowed us to identify 20 phyla, 32 classes, 62 order and 114 families. Microbial composition of S samples was compared to four fecal samples (indicated by T) obtained from loggerhead sea turtles stranded or accidentally caught along the Tuscan and Liguria coast [23].

### Diversity of bacterial communities

Estimation of rarefaction curves indicated a satisfactory level of diversity sampling (S1 Fig). Good’s coverage, used to estimate the completeness of sampling, showed a high level (0.994–0.996) in the identification of bacterial groups. Bacterial diversity estimated by the Shannon-Wiener index varied from 2.70 to 3.66 in S samples, and 2.92–4.58 in T samples, indicating similar diversity values between the two groups (Table 3). Simpson index and evenness revealed no significant difference between the two groups (S and T). Furthermore, abundance-based richness estimators, Chao1 and ACE, found in T samples a higher number of phylotypes, ranging between 203–234 than S samples, ranging from 67 to 219 (Table 3).

**Table 3:** Diversity indexes of the studied samples. Samples S are from this study, Samples T are from Abdelrhman *et al*.^23^.

Sample	Families	Good’s coverage	Chao1	ACE	α diversity	Simpson index	Shannon-Wiener diversity	Evenness
S1	26	0.996	67.42	67.35	3.50	0.07	2.781	0.853
S2	35	0.996	100.94	98.35	4.25	0.1	2.847	0.801
S3	50	0.996	133.31	128.92	3.06	0.05	3.427	0.876
S4	29	0.995	149.43	146.63	3.06	0.01	2.809	0.834
S5	38	0.996	165.39	162.45	3.05	0.05	3.284	0.902
S6	38	0.996	177.86	175.40	4.94	0.01	2.924	0.803
S7	65	0.994	189.10	188.85	3.60	0.04	3.657	0.876
S8	59	0.995	124.96	139.53	3.49	0.09	3.22	0.79
S9	42	0.996	170.45	174.54	4.69	0.13	2.70	0.72
T1	163	0.994	203.46	204.71	4.70	0.01	4.579	0.899
T3	40	0.998	211.93	213.38	5.50	0.08	2.925	0.793
T11	34	0.990	222.75	224.83	2.73	0.05	3.148	0.892
T12	34	0.993	234.39	236.02	3.97	0.05	3.074	0.871

### Taxonomic composition of the fecal bacterial communities in *C*. *caretta*

The most dominant phylum in fecal samples of *C*. *caretta* was Firmicutes with an average relative abundance of 49.4±8.0, followed by Bacteroidetes (21.5±6.3%) and Proteobacteria (11±5.3%) (Fig 1a). Less represented were Epsilonbacteraeota (2.1±1.3%) and Fusobacteria (2.1±1.3%). Bacteria belonging to other phyla (such as Synergistetes, Actinobacteria, Spirochaetes and so on) were minor components and were not present in all samples. Comparison with data from T samples revealed a similar bacterial composition, except a higher abundance of Proteobacteria in T samples (23.6±12.9%). At family level, the most dominant bacterial families were represented by Ruminococcaceae (23.8±6.4%), Rikenellaceae (10.3±3.5%), Lachnospiraceae (8.8±4.3%) and Clostridiales vadinBB60 group (6%±3%). In respect to ours, T samples were dominated by Lachnospiraceae (15.4±5.6%) Ruminococcaceae (15.3±3.6%), Clostridiaceae 1 (11.2±3.6%) and Rikenellaceae (10.2±3.7%). Both the S and T samples differed for the less represented bacterial components, as an example, Enterobacteriaceae family was found only in S5, S6, S7 and S8 samples and Flavobacteriaceae only in S3, S5 and S8.

**Fig 2:**
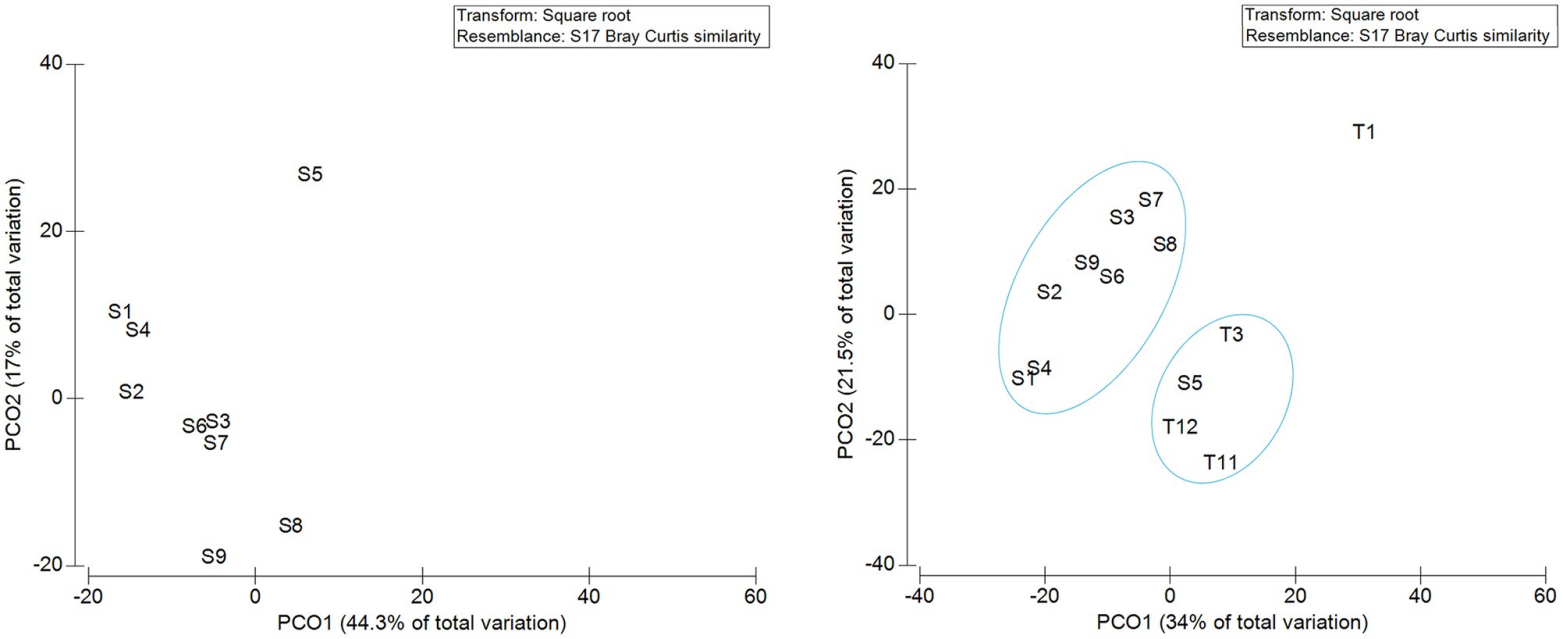
Principle coordinate analysis (PCoA) plot of S samples of this study (A) and S+T samples (B). S and T indicate samples obtained from this study and from Abdelrhman *et al*.^23^, respectively.

The PCoA plot based on Bray-Curtis distance matrix showed that most samples were dissimilar to each other with S5 clustering alone (Fig 2A). When T samples were included in this analysis, the PCoA showed that S and T samples, except S5 and T1, respectively, segregated in two independent groups. In particular, S5 and T samples cluster together; this might be due to the long period of hospitalization (S5 = 78 days; T = more than 28 days).

### Phenotypic and metabolic inference

**Fig 3:**
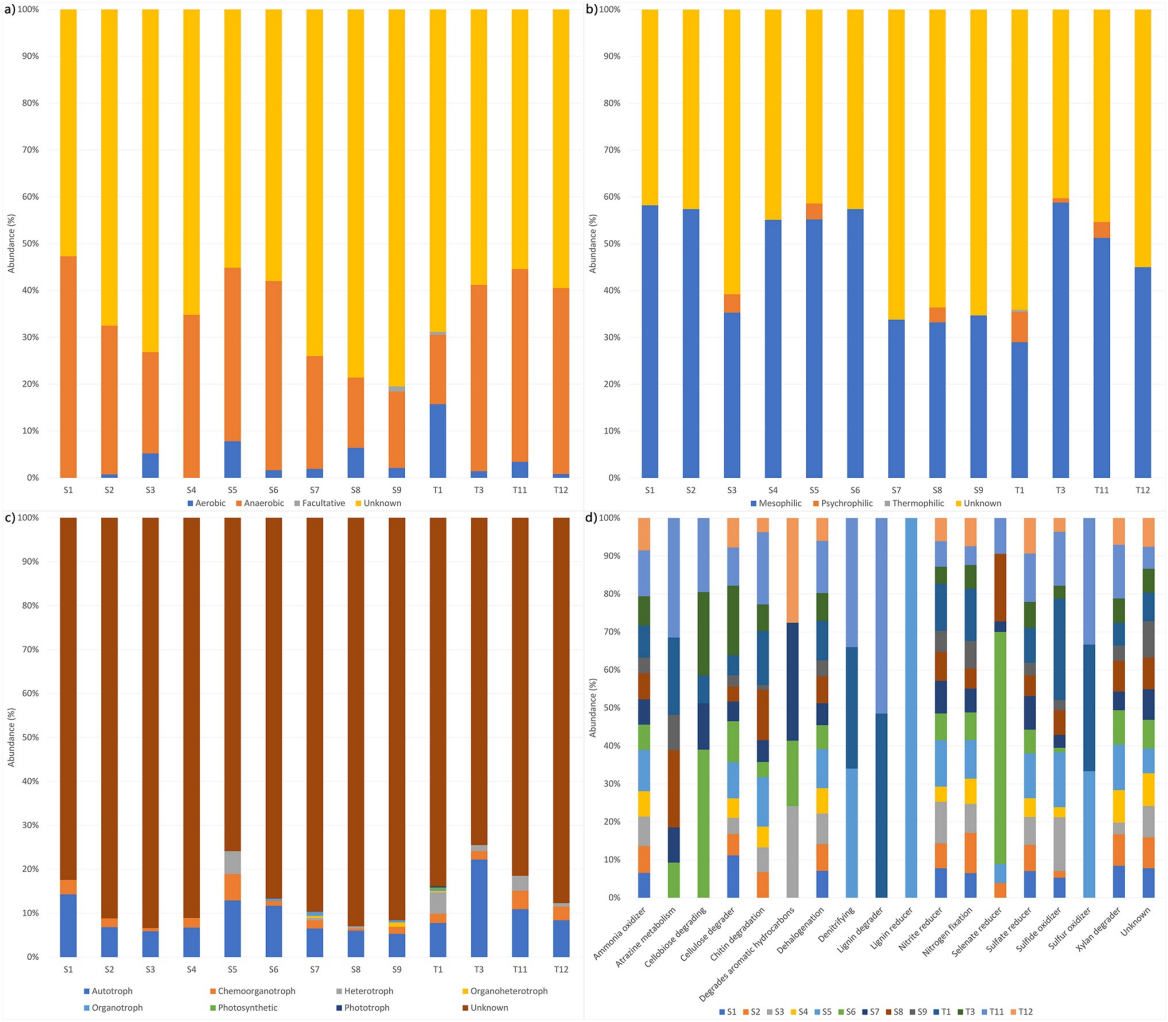
Putative metabolic requirements and activities of microbial communities of samples S and T. (A) Oxygen requirements, (B) temperature ranges, (C) energy sources, (D) type of metabolism.

Based on the inference of taxonomic-to-phenotypic mapping of metabolism using METAGENassist [35], all samples contain prevalently anaerobic and mesophilic bacteria (Fig 3A and 3B). Regarding the energy source, all samples mainly have bacteria with an autotrophic and heterotrophic metabolism (Fig 3C). Surprisingly, more differences were found when the type of metabolism was investigated (Fig 3D); in fact, all samples contain bacteria with the metabolic potential to degrade cellulose, chitin (except S1) and xylan, to reduce nitrite, and to fix nitrogen, and so on. Conversely, a few samples contain bacteria able to metabolize the pesticide atrazine (samples S6, S7, S8 and S9, T1 and T11), either to reduce selenate, a component of some pesticides (S2, S5, S6, S7, S8 and T11), or to degrade aromatic hydrocarbons (S3, S6, S7, and T12). Some samples (S5, T1 and T11) carry denitrifying and sulfur-oxidizing bacteria, whereas only samples T1 and T11 contain lignin-degraders and only S5 has lignin-reducers.

## Discussion

**Fig 4:**
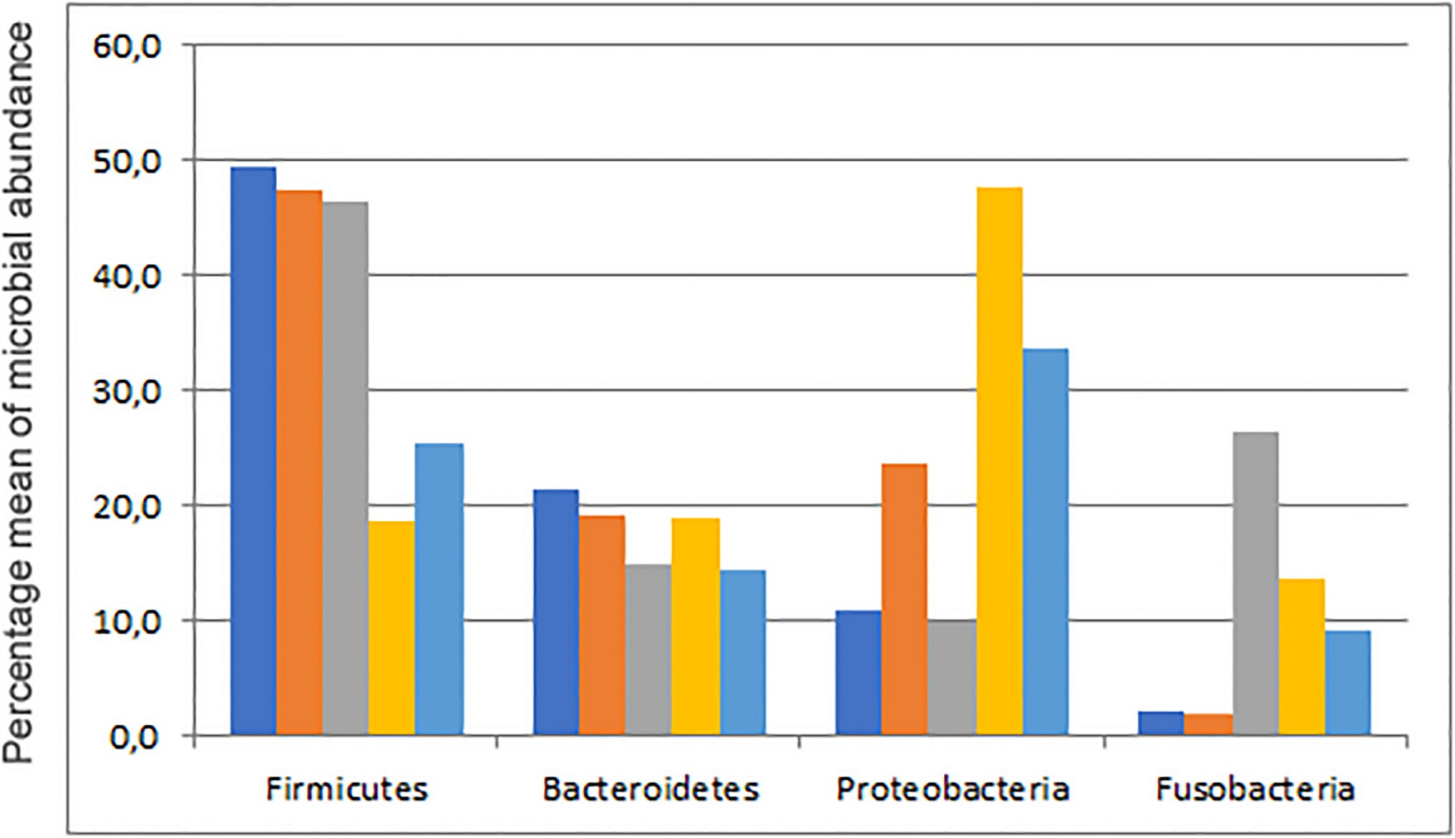
Percentage mean of abundance of main microbial components found in different studies on sea turtles. Samples are indicated as follows: blue: this study; red: Abdelrhman *et al*. [23]; grey: Biagi *et al*. [24]; yellow: Ahansan *et al*. [26]; light blue: Ahansan *et al*. [27].

In this study we aimed to expand the knowledge of the gut microbiome of the loggerhead sea turtle *Caretta caretta*. The animals were recovered and hosted after stranding along the Sicilian coast of the Mediterranean Sea. To the best of our knowledge, only a few studies have been carried out on gut microbiomes of stranded loggerhead (*C*. *caretta*) [23,24] and green (*C*. *mydas*) [26,27] sea turtles so far. Our results were compared to the above mentioned studies. The main conclusions of these studies and the corresponding microbial abundance of the four top phyla are reported in Table 4 and Fig 4. Abdelrhman *et al*. [23] and Biagi *et al*. [24] reported the fecal microbiomes of loggerhead sea turtles stranded along the Tyrrhenian and the Adriatic coast, respectively; while Ahansan *et al*. [26,27] published cloacal microbiomes of green turtles stranded along the Australian coast. Our results showed that despite the differences in origin, size and conditions of the animals, Firmicutes, Bacteroidetes, and Proteobacteria constitute the core of the gut microbiome of all stranded sea turtles. Fusobacteria are also dominant in the loggerhead sea turtles stranded along the Adriatic coast and the green turtles (Table 4).

**Table 4:** Percentage of the top four dominant phyla in the microbiome of stranded sea turtles and main features of the corresponding studies.

Sea turtle	Firmicutes	Bacteroidetes	Proteobacteria	Fusobacteria	Sample	Stranding Site	Sequenced Region	Mean days of hospitalization	Reference
*C*. *caretta*	49.4	21.5	11.0	2.1	Fecal	Sicily (Italy)	V3-V4	<13	This study
	47.4	19.0	23.6	1.9	Fecal	Tuscan and Liguria (Italy)	V4	<38	Abdelrhman, 2016
	46.5	15	10	26.5	Fecal	Adriatic coast (Italy)	V3-V4	<75	Biagi, 2018
*C*. *mydas*	18.7	19	47.6	13.6	Cloacal	Queensland (Australia)	V1-V3	AR*	Ahansan, 2017
	25.5	14.4	33.6	9.1	Cloacal	Queensland (Australia)	V1-V3	<143	Ahansan, 2018

AR * immediately after their arrival for rehabilitation.

Firmicutes represent the overwhelming majority of bacteria in all the microbiomes of *C*. *caretta* analysed so far, accounting almost for the 50% of the total microbiome (Table 4). Differently, in the fecal microbiome of the herbivorous *C*. *mydas*, Firmicutes represent the second most abundant phylum (approximately 18–25%). Firmicutes are common components found in the gut microbiota of many herbivorous reptiles [14,36–39] with the exception of the alligator, whose gut microbiome is prevalently constituted by Fusobacteria [40]. Therefore, the prevalence of Firmicutes in the gut of the herbivorous *C*. *mydas* is likely due to the diet, mostly based on seaweed. In the carnivorous *C*. *caretta* this result is somewhat surprising and it confirms that these turtles may also feed on seaweed and algae as well as wood or debris [41,42], even if in smaller quantity than on the benthic crustaceans, the sea urchins and gastropods, generally preferred by *C*. *caretta* [43–45]. Indeed, METAGEN analysis indicated that all *C*. *caretta* specimens analysed in this study contain bacteria able to degrade cellulose from different sources as well as chitin, xylan, lignin, and components of seaweed and algae. Ruminococcaceae, Rikenellaceae and Lachnospiraceae were the most dominant families, similarly to the bacterial composition found in the microbiomes of the loggerhead sea turtles analysed by Abdelrhman *et al*. [23] and of the herbivorous green turtles (*C*. *mydas)* [25,27]. Conversely, Clostridiaceae and Peptostreptococcaceae were the most represented families in the gut microbiome of the loggerhead sea turtles stranded along the Adriatic coast [24], suggesting a higher grade of dysbiosis. In the human gut Ruminococcaceae comprise “protective” intestinal bacteria while Clostridiaceae and Peptostreptococcaceae are considered harmful [46].

Besides Firmicutes, the microbial core of the microbiome of all sea turtles contains the Bacteroidetes and Proteobacteria phyla. The latter are also abundant in the human gut [47,48] as well as in other land vertebrates and reptiles [13,40]. Different Bacteroidetes/Proteobacteria ratios were determined with respect to the microbiomes of other sea turtles. In fact, our samples contained more Bacteroidetes than Proteobacteria, similarly to the results obtained in Biagi et al, while the opposite trend was registered in Abdelrhman *et al*. [23] and in stranded green turtles [26,27] (Table 4 and Fig 4). These differences could be linked to a different diet, different health conditions, or type of sample, in that Ahansan *et al*. [26,27] used cloacal swabs. Indeed, a higher abundance of Proteobacteria is recognized as a signature of dysbiosis as well as an indication of disease within the gastrointestinal tract of animals, including humans [43]. However, Proteobacteria also represent a physiologically and metabolically assorted group that can be relevant for maintaining gut pH, and for producing carbon dioxide and nutrients for further colonization by strict anaerobes. The low percentage of pathogen families found in our samples and the evidence that Proteobacteria remained the most dominant phylum even after green sea turtles rehabilitation [27] strongly suggest their role in gut homeostasis.

In contrast to the results obtained in the loggerhead sea turtles stranded along the Adriatic Coast [24] and in the green sea turtles [26,27] and similarly to the results obtained in the loggerhead sea turtles stranded along the Tuscan and Ligurian coast [23], we did not find Fusobacteria as a dominant phylum in stool samples of *C*. *caretta*. Usually Fusobacteria are scarcely abundant in reptiles [15,16,37], but can be commonly isolated from infected animals [49], and represent a dominant phylum in the microbiome of vertebrates that generally feed on carrion, i.e. alligators and vultures [40,50]. We surmise that Fusobacteria abundance increases in sea turtles after many days of hospitalization.

A comparable abundance of the phylum Bacteroidetes was found in all the microbiomes of sea turtles investigated so far. Bacteroidetes are considered commonly associated with the gut microbiota in many vertebrates. Members of the Bacteroidetes show an elaborate apparatus for acquiring and hydrolysing otherwise indigestible dietary polysaccharides. They also have an associated environment-sensing system consisting of a large repertoire of extracytoplasmic function sigma factors and signal transduction systems. Thus, the enzymatic and regulatory activities of Bacteroidetes may contribute to the turtle adaptation to the digestion of accidentally ingested food containing carbohydrates. [5,51].

Gut microbiome was not found to be related with the curved carapace length in accordance with results reported in *C*. *mydas* [25–27] and in contrast with the report on the loggerhead sea turtles stranded along the Adriatic coast [24]. The results obtained by Biagi *et al*.[24] could reflect an adaptation of microbiota to the diet and housing conditions at the recovery centre since most samples were collected after many days of hospitalization (up to 240 days).

PCoA and diversity indices showed heterogeneity between fecal samples of this study collected after a few days (2–7) and many days (more than 28) of hospitalization, independently of the stranding location, suggesting that hospitalization and diet could influence gut microbiota. This result is in accordance with the reports on *C*. *mydas* [25–27] and in contrast with results obtained on *C*. *caretta* stranded along the Adriatic coast [24].

Surprisingly, bacteria capable of metabolizing pesticides, like atrazine and sodium selenate, were found in our samples suggesting that these compounds are present in the Mediterranean Sea. Despite its EU-wide ban in 2004, the pesticide atrazine is frequently detected in the aqueous environment [52]. In addition, ammonia-oxidizers and bacteria capable of dehalogenate organic compounds were found in all the analysed samples. Considerable amounts of ammonia are usually present in sewage treatment plants and both haloaliphatic and haloaromatic compounds are produced industrially in large quantities and represent an important class of environmental pollutants [53]. These bacteria may have been ingested through accidentally contaminated food or sediment or sea water. It remains to be investigated whether the gut microbial community is modified after the ingestion of pollutants, since we were not able to determine if the bacteria are transiently or stably associated with the sea turtle gut.

Moreover, we cannot exclude that microbiome differences could be related to the origin of the sample, the time of sample collection, or to diseases, stress or other processes that influence the immune system, as demonstrated in other reptiles [54]. Finally, our data indicates that the 8% of the total bacteria were not identified, revealing that many classes and their metabolic capabilities are still to be unveiled.

## Supporting information

S1 Fig Rarefaction curves on total filtered sequencing data of *Caretta caretta* fecal microbiota. (TIF)

S2 Fig Matrix of the bacteria present in the nine samples. Blue boxes indicate the presence.
(TIF)

S1 Table Total number of OTUs resulting from the QIIME pipeline. (DOCX)
