# Supplementary figures and images for: New insights into the gut microbiome in loggerhead sea turtles Caretta caretta stranded on the Mediterranean coast
Source: PLoS One. 2019 Aug 14;14(8):e0220329. doi: 10.1371/journal.pone.0220329 (PMC6693768; doi:10.1371/journal.pone.0220329)

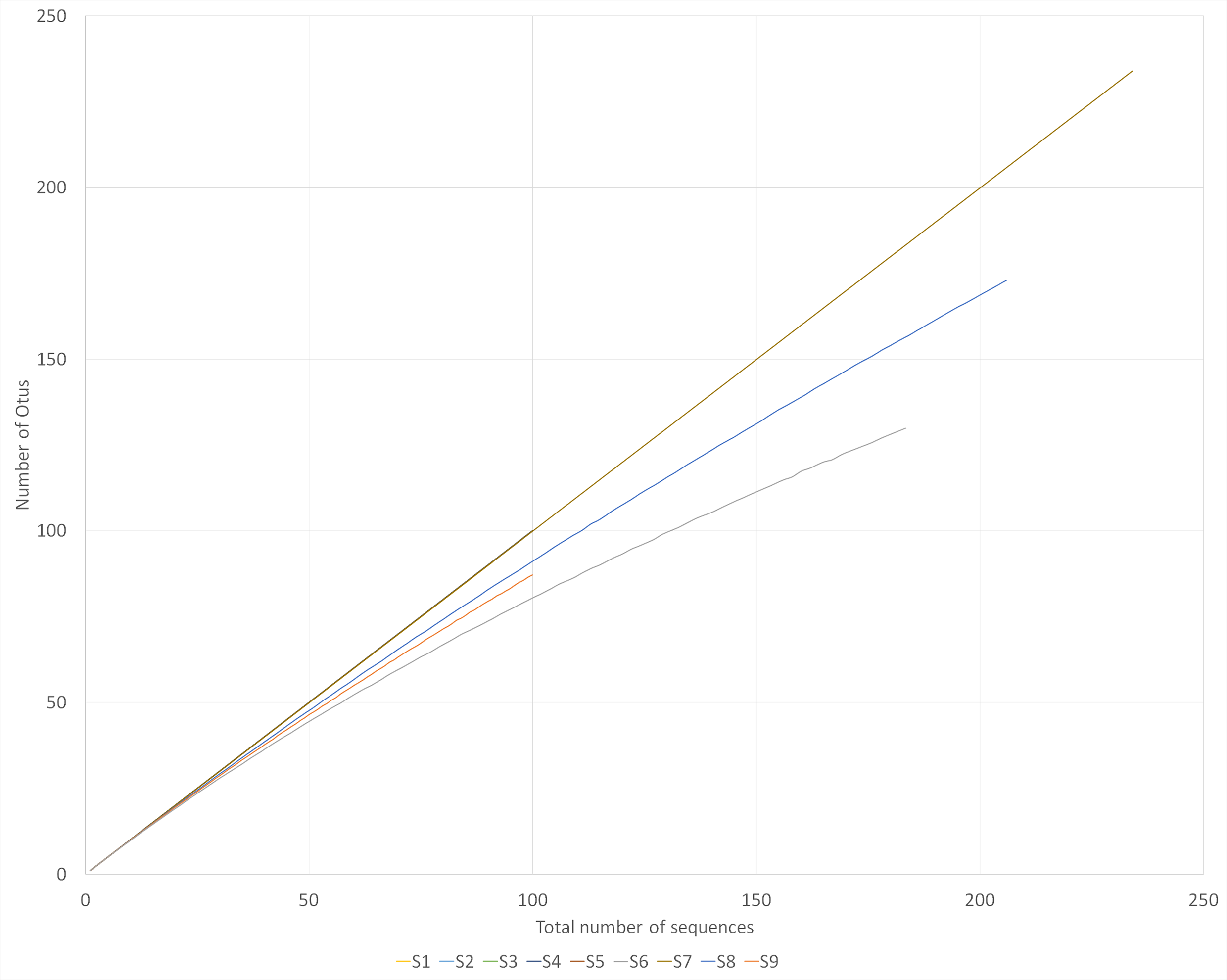

Supplement: S1 Fig — (TIF) [file pone.0220329.s001.tif]

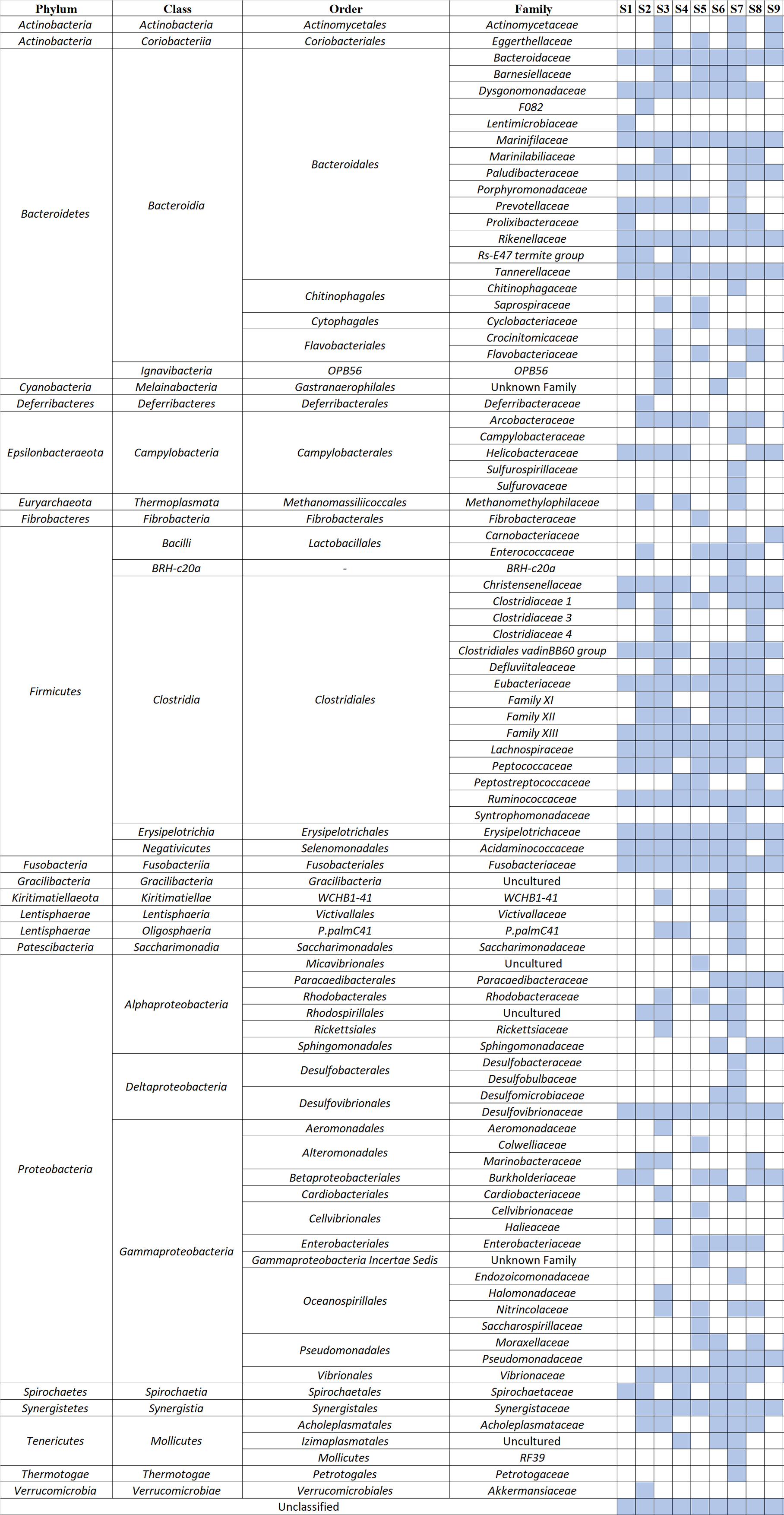

Supplement: S2 Fig — Blue boxes indicate the presence. (TIF) [file pone.0220329.s002.tif]
