# Supplementary material for: New insights into the gut microbiome in loggerhead sea turtles Caretta caretta stranded on the Mediterranean coast
Source: PLoS One. 2019 Aug 14;14(8):e0220329. doi: 10.1371/journal.pone.0220329 (PMC6693768; doi:10.1371/journal.pone.0220329)
Supplement: S1 Table — (DOCX) [file pone.0220329.s003.docx]

| **Sample** | **Total Reads** | **Filtered Reads** | **OTUs** |
| --- | --- | --- | --- |
| S1 | 99926 | 10772 (10.7%) | 96 |
| S2 | 129304 | 16179 (12.5%) | 126 |
| S3 | 166807 | 26925 (16.1%) | 138 |
| S4 | 102189 | 12775 (12.5%) | 115 |
| S5 | 130991 | 35705 (27.3%) | 196 |
| S6 | 140362 | 21322 (15.2%) | 151 |
| S7 | 144340 | 39998 (27.7%) | 243 |
| S8 | 48370 | 8416 (17.4%) | 211 |
| S9 | 55625 | 12849 (23.1%) | 202 |
| Total OTUs |  |  | 1478 |
